# Self-medication with Antibiotics in WHO Southeast Asian Region: A Systematic Review

**DOI:** 10.7759/cureus.2428

**Published:** 2018-04-05

**Authors:** Gaurav Nepal, Shekhar Bhatta

**Affiliations:** 1 Maharajgunj Medical Campus, Tribhuvan University Institute of Medicine

**Keywords:** antibiotics, self-medication, southeast asia

## Abstract

Antibiotics are essential treatments, especially in the developing world like World Health Organization (WHO) Southeast Asian region where infectious diseases are still the most common cause of death. In this part of the world, antibiotics are purchased and used without the prescription of a physician. Self-medication of antibiotics is associated with the risk of inappropriate drug use, which predisposes patients to drug interactions, masking symptoms of an underlying disease, and development of microbial resistance. Antibiotic resistance is shrinking the range of effective antibiotics and is a global health problem. The appearance of multidrug-resistant bacterial strains, which are highly resistant to many antibiotic classes, has raised a major concern regarding antibiotic resistance worldwide. Even after decades of economic growth and development in countries that belong to the WHO Southeast Asian region, most of the countries in this region still have a high burden of infectious diseases. The magnitude and consequence of self-medication with antibiotics is unknown in this region. There is a need for evidence from well-designed studies on community use of antibiotics in these settings to help in planning and implementing specific strategies and interventions to prevent their irrational use and consequently to reduce the spread of antibiotic resistance. To quantify the frequency and effect of self-medication with antibiotics, we did a systematic review of published work from the Southeast Asian region.

## Introduction and background

Antibiotics are among the most commonly purchased drugs worldwide [1]. They are essential treatments, especially in the developing world where infectious diseases are still the most common cause of death [2]. Self-medication refers to the use of medicines to treat self-diagnosed disorders without consulting a medical practitioner and without any medical supervision [3]. It is a form of healthcare practiced in most parts of the world and overall 50% of total antibiotics used are purchased over-the-counter [4-5]. Repercussions of self-medication with antibiotics leading to health hazards, particularly in the developing world, are multifaceted as they are linked to poverty, inaccessibility, lack of medical professionals, poor quality of healthcare facilities, unregulated distribution of medicines, and patients’ misconceptions about physicians [6-7].

Self-medication of antibiotics is associated with the risk of inappropriate drug use, which predisposes patients to drug interactions, masking symptoms of an underlying disease, and the development of microbial resistance [8-9]. The inappropriate drug use practices common in self-medication include short duration of treatment, inadequate dose, sharing of medicines, and avoidance of treatment upon the improvement of disease symptoms [10]. The appearance of multidrug-resistant bacterial strains, which are highly resistant to many antibiotic classes, has raised a major concern regarding antibiotic resistance worldwide. This resistance may result in prolonged illnesses, more doctor visits, extended hospital stays, the need for more expensive medications, and even death [11].

Although various individual studies have examined antibiotic self-medication in countries that belong to the World Health Organization Southeast Asia region (WHO SEAR), there has not been a systematic review done in this setting. Even after decades of economic growth and development in countries that belong to the WHO SEAR, most of the countries in this region still have a high burden of infectious diseases [12]. There is a need for evidence from well-designed studies on the community use of antibiotics in these settings to help in planning and implementing specific strategies and interventions to prevent their irrational use and consequently to reduce the spread of antibiotic resistance. To quantify the frequency and effect of self-medication with antibiotics, we did a systematic review of published work from WHO SEAR.

## Review

Methods

Search Strategy

Databases (PubMed, PubMed Central, and Google Scholar) were searched for peer-reviewed research published between January 2000 and January 2018. The search terms, viz. antimicrobial, antibiotics, antibacterial, self-medication, and non-prescription combined with the name of countries that belong to the WHO SEAR, were used. Medical subject headings (MeSH) of the search terms were used in each case to maintain common terms across all databases searched. A thorough review of the references revealed further relevant articles.

Selection Criteria

**Figure 1:**
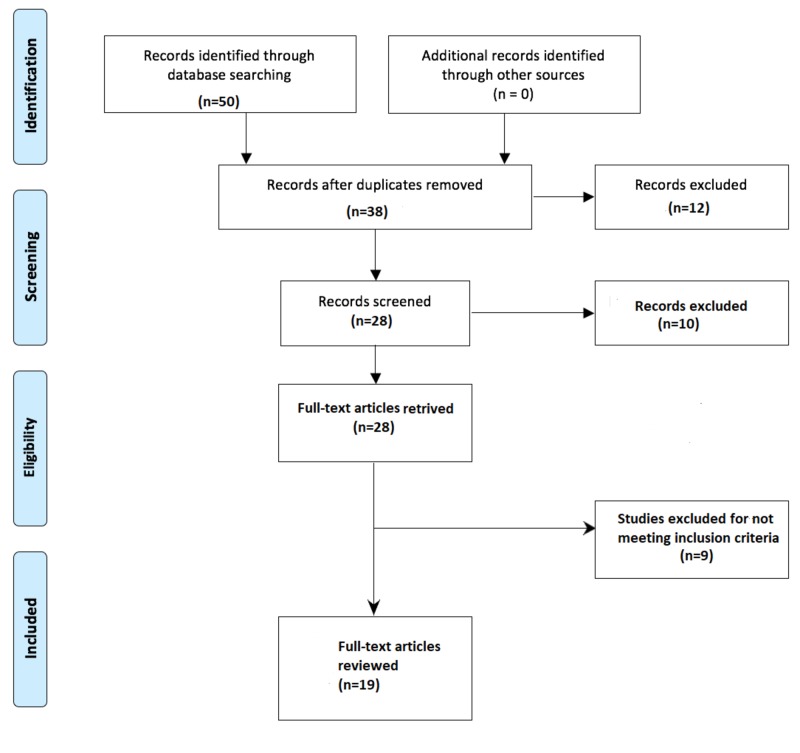
PRISMA diagram detailing the study identification and selection process PRISMA: Preferred Reporting Items for Systematic Reviews and Meta-Analyses

Studies published in the English language were included in the review if they aimed to assess self-medication of antibiotics in countries that belong to WHO SEAR. Studies on antivirals, antifungals, antiprotozoal, and topical antimicrobials were excluded. In addition, studies dealing with self-medication of overall drugs, editorials, correspondences, and letters to the editor were also debarred. A Preferred Reporting Items for Systematic Reviews and Meta-Analyses (PRISMA) diagram detailing the study identification and selection process is given in Figure 1.

Data Abstraction

The authors screened the articles based on the inclusion/exclusion criteria. Full texts were obtained for articles that met inclusion criteria. Authors developed a data abstraction spreadsheet using Microsoft Excel version 2013 (Microsoft Corp., Redmond, WA, USA) and included the following information: author, year of publication, journal, country where the study was done, recall period, study design, sample size, population sampled, prevalence of antimicrobial self-medication, type of antimicrobial agents used, source of drugs, disease symptoms, and inappropriate drug use practices.

Results

Study Selection

The initial electronic search identified 50 articles. After adjustment for duplicates, 38 remained. Of these, 10 studies were discarded, since, after review of their titles and abstracts, they did not meet the criteria. The full texts of the remaining 28 studies were reviewed in detail. Nine studies were cast away after the full text had been reviewed since they did not address much of the needed information. Finally, 19 studies were included in the review. A PRISMA diagram detailing the study identification and selection process is given in Figure 1.

Study Characteristics

**Figure 2:**
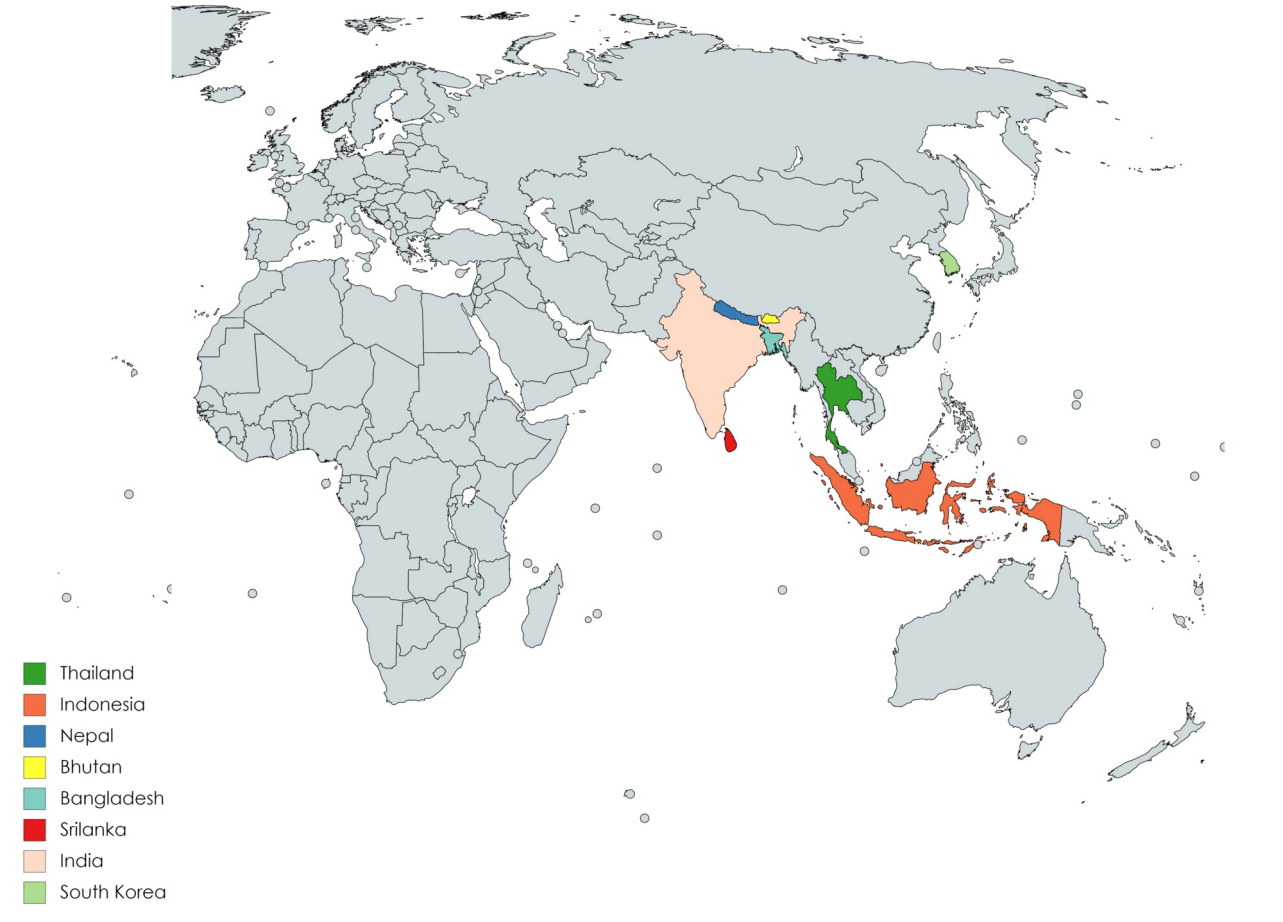
Countries included in this study

Almost all 19 studies differed in their setting, recall period, sample size, and study subjects. The studies covered 11,197 participants and the sample size ranged from 110 to 2,996. All studies included in this review were cross-sectional surveys. The studies were performed in WHO SEAR (Bhutan, Bangladesh, India, Indonesia, South Korea, Nepal, Srilanka, and Thailand) and illustrated in Figure 2. No studies were available from three countries of WHO SEAR (Myanmar, Maldives, and Timor-Leste). The recall period used in data collection varied among the different studies, ranging from one month to one year. A recall period was not available for all included studies. Studies were conducted among the general public, university students, and medical professionals. A detailed description of the characteristics of individual studies is provided in Table 1.

**Table 1:** Key Characteristics of Included Studies NA: not available; SMA: Self medication with antibiotics

Study	Country	Year	Design	Recall time	Sample size	Subjects	SMA Prevalence (%)
Tshokey et al. [13]	Bhutan	2017	cross-sectional survey	NA	692	General public	23.6%
Biswas et al. [14]	Bangladesh	2014	cross-sectional survey	3 months	1300	General public	26.69%
Seam et al. [15]	Bangladesh	2018	cross-sectional survey	NA	250	Pharmacy students	15.6%
Shubha et al. [16]	India	2013	cross-sectional survey	NA	110	Dentists	78.18%
Biswas et al. [17]	India	2015	cross-sectional survey	6 months	164	Nursing students	54.2%
Nair et al. [18]	India	2015	cross-sectional survey	1 year	221	Medical students	85.59%
Ahmad et al. [19]	India	2012	cross-sectional survey	NA	600	General public	33.5%
Pal et al. [20]	India	2016	cross-sectional survey	NA	216	Medical and pharmacy students	75%
Virmani et al. [21]	India	2017	cross-sectional survey	1 years	456	Health science students	60%
Ganesan et al. [22]	India	2014	cross-sectional survey	NA	781	General public	39.4%
Widayati et al. [23]	Indonesia	2011	cross-sectional survey	1 month	559	General public	7.3%
Hadi et al. [24]	Indonesia	2008	cross-sectional survey	1 month	2996	General public	16%
Kurniawan et al. [25]	Indonesia	2015	cross-sectional survey	6 months	400	General public	45%
Kim et al. [26]	Korea	2011	cross-sectional survey	NA	1,177	General public	46.9%
Sah et al. [27]	Nepal	2016	cross-sectional survey	NA	327	Nursing students	50.7%
Pant et al. [28]	Nepal	2015	cross-sectional survey	1 year	168	Dental students	35.1%
Banerjee et al. [29]	Nepal	2016	cross-sectional survey	NA	488	Medical students	26.2%
Rathish et al. [30]	Sri Lanka	2017	cross-sectional survey	1 month	696	Medical students	39%
Sirijoti et al. [31]	Thailand	2014	cross-sectional survey	3 months	396	General public	37.37%

Prevalence of Self-Medication

The prevalence of self-medication with antibiotics (SMA) ranged from 7.3% to 85.59% with an overall prevalence of 42.64%. Prevalence rates differed greatly between countries and study subjects, as is summarized in Table 1. A high prevalence was reported from India and Nepal, and a low prevalence was reported from Indonesia and Bangladesh. The prevalence of SMA was higher among men in most studies. The prevalence of SMA was higher among health students and health professionals and was low among the general public.

Common Illnesses and Reasons that Led to Self-Medication

The common cold, sore throat, fever, gastrointestinal tract diseases, and respiratory diseases were the commonest illnesses or symptoms for which self-medication was taken. The major reasons behind the frequent practice of SMA were prior experiences of treating a similar illness, ignorance regarding the seriousness of the disease, an assured feeling of not requiring a visit to the physician, less expensive and easily affordable in terms of time and money, knowledge of the antibiotics, and suggestions from others. Table 2 shows the illnesses that resulted in self-medication and the reasons that drove people to practice self-medication as reported in each study.

**Table 2:** Illnesses and Reasons for Self-medication with Antibiotics NA: not available; OPD: outpatient department; GIT: gastrointestinal tract

Study	Illnesses	Reasons
Tshokey et al. [13]	NA	NA
Biswas et al. [14]	GIT problems (36.02%) Cold, cough and fever (28.24%) Infection (12.97%)	Pre-experience (45.82%) Suggestions from others (28.24%) Knowledge of the antibiotics (16.14%) Reduction of doctor’s fees (6.34%) No confidence with doctor’s medication (3.46%)
Seam et al. [15]	NA	Old prescription Academic knowledge Internet Advertisement Friends
Shubha et al. [16]	Sore throat (44.19%) Common cold (41.86%) Toothache (39.53%) Diarrhea (24.42%) Flu (9.30%) Other respiratory conditions (5.81%)	Being a dentist (40%) previous prescription (16.87%) Considering the symptoms as minor (14.46%)
Biswas et al. [17]	Common-cold Cough Sore throat, diarrhea, fever Burning micturition Skin infections	NA
Nair et al. [18]	Common cold, cough, and sore throat (62.4%) Fever (25.3%) Gastrointestinal tract infections (18.6%)	Easier to apply previous prescription (46.6%) Convenience (21.7%) Good knowledge of antibiotics (11.3%)
Ahmad et al. [19]	Respiratory tract infection (16.83%), wound infection (14.5%) Cough and cold (14.5%) Gastrointestinal tract infection (13.66%) Fever (13.66%) Skin disorders (13%) Eye and ear infections (7%), Acne (2.33%), Urinary tract infections (1%) Other diseases (3.5%)	Disease is simple (21.83%) Treatment cost is high in hospitals (30%) Previous experience with the disease (8.16%) Lack of hospitals in the nearest place (8.16%) Patient knows about the drug and disease (11.5%) Lack of trust in medical service (4%)
Pal et al. [20]	Medical students: fever (48.4%), diarrhea (47.6%), cough (46%), sore throat (43.6%) Pharmacy students: fever (46.8%), cough (30.6%), sore throat (29.7%), and diarrhea (23.4%)	Sufficient pharmacological knowledge Timesaving Avoiding crowd at OPD Cost saving
Virmani et al. [21]	Middle ear infection Clear nasal discharge Purulent nasal discharge Sore throat Flu-like symptoms Skin infections	NA
Ganesan et al. [22]	Coughs and sore throat Cold and fever Ear infections Flu conditions Toothache	NA
Widayati et al. [23]	Common-cold, including cough, sore throat, headache, and other minor symptoms	Previous experience Saving time Saving money
Hadi et al. [24]	NA	NA
Kurniawan et al. [25]	Wounds or skin diseases (32.2%) Acute respiratory infections (18.3%) Fever (11.7%)	More practical than seeking a doctor Too busy to see a doctor Previous experience No money to pay for doctor
Kim et al. [26]	NA	NA
Sah et al. [27]	Fever (35.7%) Sore throat (20.2%) Rhinitis (12.5%)	Good knowledge of antibiotic (46.2%) Doctor advice is not needed for common illness (32%) To save time and money (21.3%)
Pant et al. [28]	Fever (39.0%) followed by sore throat, cough, diarrhoea, and runny nose	Previous prescriptions (42.4% ) Recommended by pharmacists (37.3%) Own experience (25.4%) Opinion of family and friends (18.7%)
Banerjee et al. [29]	NA	NA
Rathish et al. [30]	Sore throat (46%): most common Runny nose, Flu Diarrhea	Previous experience No access to physician care
Sirijoti et al. [31]	NA	Buy antibiotics yourself by bringing old antibiotics packaging or the sample of used antibiotics Buy antibiotics yourself by suggestions from your friends and family Advertisement

Source of Medicines

The majority of the antimicrobial drugs used in self-medication were obtained from various sources, such as pharmacies, leftover drugs, hospitals, and from friends and family. The use of self-medication was commonly suggested by pharmacy professionals, friends, family, and relatives among the general public, whereas among health students and health professionals, self-medication was because of knowledge of medicine and pharmacology.

Antibiotics Used in Self-Medication

The most common antibiotic used for self-medication was amoxicillin, followed by macrolides, fluoroquinolones, cephalosporins, and metronidazole [14, 16-25, 27-30]. Antibiotics used for self-medication in each of the included studies are given in Table 3. Of the 19 studies included in the review, four did not investigate the types of antibiotics used in self-medication [13, 15, 26, 31]. Among the macrolides, azithromycin use was most common, and among the fluoroquinolones, ciprofloxacin use was most common.

Inappropriate Use of Antibiotics

Only seven studies included in the review reported the inappropriate use of antibiotics [13, 16, 20-21, 26, 28, 31]. The most inappropriate practice was an abrupt stoppage of the antibiotic course after the disappearance of symptoms. Other improper practices were sharing antibiotics, saving antibiotics for future use, and switching antibiotics if symptoms were not relieved.

**Table 3:** Antibiotics Used in Self-medication, Inappropriate Use, and Source NA: not available

Study	Inappropriate drug use	Most common antibiotics used	Source of drugs
Tshokey et al. [13]	13.1% shared antibiotics with other people 19.0% saved antibiotics for later use	NA	NA
Biswas et al. [14]	NA	Metronidazole (50.43%) Azithromycin (20.75%) Ciprofloxacin (11.53%) Amoxicillin (10.37%) Tetracycline (7.49%)	Pharmacies
Seam et al. [15]	NA	NA	Pharmacies
Shubha et al. [16]	Switch-over of antibiotics inbetween (12.79%) Abrupt stoppage (15.12%)	Amoxicillin (70.93%) Azithromycin (20.06%) Ciprofloxacin (8.14%) Metronidazole (5.81%) Ofloxacin (4.65%)	Medicine at home/clinic
Biswas et al. [17]	NA	Metronidazole (67.4%) Azithromycin (32.6%) Norfloxacin (16.8%)	Leftover medicines at home Pharmacies
Nair et al. [18]	NA	Azithromycin (34.4%) Amoxicillin (29.4%) Fluoroquinolones (18.6%)	Pharmacies Left-over drugs from previous prescriptions Family/friends
Ahmad et al. [19]	NA	24.16% cephalosporin 21.16% of penicillin 16.16% of quinolone Tetracycline group (12.83%) Sulfonamides (10.16%) Macrolide groups of antibiotic (4.83%) Aminoglycoside (3.16%) Metronidazole (3.33%)	Family, friends, and neighbors Pharmacies Previous prescription
Pal et al. [20]	Only 72.2% of medical students and 33.3% of pharmacy students took full course of antibiotics	Amoxicillin Azithromycin	NA
Virmani et al. [21]	Very few completed the course once started	B-Lactams (Most common) Fluoroquinolones Macrolides Tetracycline	NA
Ganesan et al. [22]	NA	Amoxicillin (most common) Erythromycin Cephalosporin Azithromycin Norfloxacin	Pharmacies Friends Old prescription
Widayati et al. [23]	NA	Amoxicillin (most common) Ampicillin Fradiomisin-gramicidin Tetracycline ciprofloxacin	Pharmacies Drug stores Kiosks
Hadi et al. [24]	NA	Amoxicillin or ampicillin (most common) Tetracycline Cotrimoxazole Chloramphenicol Thiamphenicol	Pharmacies Drugstores Friends and relatives kiosks
Kurniawan et al. [25]	NA	Amoxicillin (68.3%) Ampicillin (26.1%) Cefadroxil (1.1%) Others (5.3%)	Pharmacies Stalls Leftover antibiotics Friends or family
Kim et al. [26]	77.6% of respondents stopped taking the medication when they felt better	NA	NA
Sah et al. [27]	NA	Amoxicillin (33.9%) Azithromycin (14.9%) Ciprofloxacin (13.7%)	NA
Pant et al. [28]	Switched antibiotics Stopped the antibiotic use after the disappearance of the symptoms Stopped before finishing the course in a few days regardless of the outcome	Amoxicillin (most common) Metronidazole Azithromycin Ciprofloxacin Amoxicillin and clavulanic combination Cephalexin Ofloxacillin	Pharmacies
Banerjee et al. [29]	NA	Amoxicillin Azithromycin	Pharmacies
Rathish et al. [30]	NA	Amoxicillin (most common) Ciprofloxacin Co-amoxiclav Tetracycline Azithromycin Ciprofloxacin Cephalosporin	Pharmacies Relatives or friends Leftover drugs at home
Sirijoti et al. [31]	Distribute to another person who has the same symptoms Stop taking antibiotics as soon as symptoms are relieved Stock some antibiotics at home in case of emergency	NA	Leftover drugs at home Pharmacies

Discussion

The main finding of this review is that there are many published studies to indicate that the prevalence of SMA is alarmingly high among member countries of WHO SEAR. The prevalence of self-medication varied across the studies reviewed, ranging from 7.3% to 85.59%, with an overall prevalence of 42.64%. The main reasons for the wide variation in the prevalence of the self-medication practice may be differences in social determinants of health, tradition, culture, economic status, and developmental status. The difference in methodology, study setting, sample population, and recall time may also have contributed to this variation in prevalence of self-medication. A systematic review by Alhomoud et al. reported that the overall prevalence of self-medication varied from 19% to 82% in the Middle East [32]. A similar review by Ayalew et al. found that the prevalence of self-medication varied across the studies, ranging from 12.8% to 77.1% in Ethiopia [33]. The results of the current review are similar to those reported for SMA in the Euro-Mediterranean region [34] and developing countries [35]; the overall median proportions of self-medication reported for these countries were 40.9% and 38.8%, respectively. Developed countries, such as those of Europe where over-the-counter antibiotic sales are strictly regulated, have much lower prevalence rates of SMA, ranging from 1% to 4% [36].

Comparatively, higher self-medication use was reported in studies conducted on health science students than the general public. This may be because of the better understanding of disease and drugs leading to a decreased inclination towards seeking physicians’ help to treat their illnesses. Other studies conducted on health science students in different parts of the world have also reported a higher prevalence of self-medication practice [37-38]. Previous experience of treating a similar illness, feeling that the illness was mild and did not require the service of a physician, less expensive in terms of time and money, gaps in terms of knowledge, attitudes, and practices regarding antibiotic use, such as keeping leftover antibiotics for future use, sharing antibiotics with others, and belief that antibiotics can speed up recovery and eradicate any infection, were the most common reasons for SMA among the general public.

This review found that the main source of antibiotics used for self-medication were pharmacies, followed by friends and family. Pharmacists often do not have an adequate knowledge of the antimicrobial agents and the disease processes. However, they are commonly preferred as a source of advice or information for the antimicrobial agents obtained and used over-the-counter. Thus, pharmacists could play an important role in educating patients, rationalizing antibiotic use, and stopping antibiotic sales without a prescription.

Settings in which individuals are highly educated tend to have relatively low levels of use of antimicrobial self-medication. Therefore, awareness among communities is an important target to minimize antimicrobial self-medication in WHO SEAR. Due to their prior successful use of antimicrobial agents, individuals in most communities tend to believe that they can manage subsequent illnesses without consulting a physician. This is a potential risk factor for inappropriate drug use since most patients lack knowledge of the disease process and the medicines used in self-medication. The reasons for self-medication with antibiotics are different according to settings and are due to the complex network of a poor health system, social, economic, and health factors [39]. Therefore, establishing these factors is of paramount importance in designing and implementing programs against self-medication with antibiotics.

The underlying challenges of health systems in most countries of WHO SEAR, such as inadequate healthcare, potentially influence the use of self-medication [39]. In addition, the lack of policies or their inadequate implementation enables easy over-the-counter access of antibiotics [40]. Furthermore, most developing countries face the challenge of an irregular supply of drugs to the public health facilities, which limits community access to healthcare. This, coupled with the high burden of infectious diseases in these countries, makes the private sector an important alternative source of healthcare [41].

The common cold, sore throat, fever, gastrointestinal tract diseases, and respiratory diseases were the commonest illnesses or symptoms for which self-medication was taken. Fever and cold were indicated as the most frequent health complaint that led to self-medication in different studies [42-43]. There were also studies that reported respiratory diseases [44] and gastrointestinal (GI) tract diseases [45] as common illnesses for which self-medication was used. This may be because these illnesses are very common and occur frequently in individuals with experience in treating them. The mild and self-limiting nature of these illnesses may also prevent patients from seeking physician consultation. However, patients should not forget that when these illnesses/symptoms occur repeatedly or for prolonged periods, they should be investigated further by physicians, as they may be manifestations of serious illnesses.

Self-medication with antibiotics occurred with different antibiotic classes. The most common antibiotics used for self-medication was amoxicillin, followed by macrolides, fluoroquinolones, cephalosporins, and metronidazole. The high use of amoxicillin and fluoroquinolones may be due to the low cost, easy availability, and low side effect profiles. Amoxicillin, fluoroquinolones, and macrolides are also the most commonly prescribed antibiotics in this region and patients tend to use these prescriptions as a reference for similar illness in future [46-47]. Amoxicillin is a useful first-line antibiotic for acute otitis media, pneumonia, urinary tract infections, and other infections. Rampant, irrational use leads to resistance and treatment failure. Drugs from the quinolone group of antibiotics are reserved as second-line drugs for tuberculosis. Self-medication and inappropriate use of ciprofloxacin make people vulnerable to drug-resistant tuberculosis.

The review established an inappropriate practice of antibiotic self-medication in communities of WHO SEAR. The most common inappropriate practice was an abrupt stoppage of a course of antibiotics after the disappearance of the symptoms. Another inappropriate practice was sharing antibiotics, saving antibiotics for future use, and switching antibiotics if symptoms were not relieved. However, the clinical outcomes of antibiotic self-medication were rarely reported in the articles from most studies in the WHO SEAR, probably because of a lack of awareness about the potentially harmful effects of antibiotics. These inappropriate uses potentially increase the risk of mistreatment, adverse drug reactions, drug interactions, and the development of resistance.

Some studies included in the review reported self-medication using multiple antimicrobial agents. The use of more than one antibiotic during an illness episode is indicative of the uncertainty of the cause of illness. These inappropriate practices potentially increase the risk of mistreatment, adverse drug reaction, development of resistance and drug interactions [8, 41]. This is further worsened by the high burden of infectious diseases in addition to the limited therapeutic choices in most WHO Southeast Asian countries [41]. Antibiotic resistance is likely to add further financial strains to the healthcare system, which currently is already facing the challenge of inadequate funding. This is especially the case as patients with resistant infections are likely to stay longer in hospitals and there is a need for more expensive second-line antibiotics. Agencies, such as the World Health Organization (WHO), the South Asian Association for Regional Cooperation (SAARC), the Association of Southeast Asian Nations (ASEAN), and the Ministry of Health of countries belonging to WHO SEAR, need to establish specific interventions focusing on these common inappropriate antibiotic use practices.

Thus, the situation can be changed in the WHO SEAR by enforcing and controlling laws and regulations related to the antibiotic dispensation in pharmacies and by increasing public awareness about the adverse drug reactions, development of superinfections, and antibiotic-resistance. These problems require appropriate measures by policymakers to develop pertinent policies, as well as to ensure their implementation.

## Conclusions

The prevalence of SMA is comparatively high in the countries of WHO SEAR and is marked with inappropriate use of drugs, which is the leading cause of antibiotic resistance. Educational interventions targeting the general public, pharmacists, and healthcare students are of utmost importance. In addition, the improvement in the quality of healthcare facilities with easy access, law enforcement, and control regulations regarding the inappropriate use of antibiotics closely collaborating with public awareness about antibiotic resistance could alleviate and, ultimately, eradicate the challenge of SMA in this region. Since many patients get knowledge about drugs from the previous prescriptions, physicians should limit superfluous prescriptions of antibiotics and implement guideline-based practices. Pharmacists should also be morally encouraged to educate patients and rationalize antibiotic use by strictly stopping antibiotic sales without an authorized prescription by physicians.
